# Pharmacological Potential of Cafestol, a Bioactive Substance in Coffee, in Preventing Ischemia-Reperfusion-Induced Acute Kidney Injury

**DOI:** 10.1021/acsomega.4c11728

**Published:** 2025-05-27

**Authors:** Dayene S. Gomes, Mayara A. Romanelli, Stela P.S. Gomes, Ana Laura M. Brand, Rodrigo M.V. da Silva, Simone S.C. Oliveira, André L.S. Santos, Claudia M. Rezende, Lucienne S. Lara

**Affiliations:** a Instituto de Ciências Biomédicas, Universidade Federal do Rio de Janeiro, Rio de Janeiro, RJ 21941-902, Brazil; b Centro de Pesquisa em Medicina de Precisão, 28125 Universidade Federal do Rio de Janeiro, Rio de Janeiro, RJ 21941-902, Brazil; c Instituto de Química, Centro de Ciências Matemáticas e da Natureza, 28125 Universidade Federal do Rio de Janeiro, Rio de Janeiro, RJ 21941-909, Brazil; d Instituto de Microbiologia Paulo de Góes, Universidade Federal do Rio de Janeiro, Rio de Janeiro, RJ 21941-902, Brazil

## Abstract

Renal ischemia-reperfusion (I/R) is the leading cause of acute kidney injury (AKI) and is a relevant complication of kidney transplantation. This work evaluated whether a single dose of cafestol (CAF) prevents I/R-induced AKI. We randomly divided male Wistar rats (180–220 g) into sham-operated groups (CTRL) or I/R surgery groups (I/R), which received either CAF 50 mg/kg or 75 mg/kg orally 2 h before the I/R procedure. During 24 h of reperfusion, rats were placed in metabolic cages for urine collection. The rats were euthanized, blood and urine were stored for renal function analyses, and the kidneys were collected for biochemical and histological studies. The I/R rats developed AKI, as evidenced by kidney damage (space between tubules, glomerular segmentation, and marked collagen accumulation throughout the renal tissue) and a decline in function and Na^+^ handling: proteinuria (120% increase), elevated plasma urea nitrogen (40%) and plasma creatinine (66%), and reduced fractional Na^+^ excretion (39%). These observations were associated with a 52% reduction in HIF-1α protein content and a 357% increase in matrix metalloproteinase (MMP)-9 activity. CAF decreased the urine volume and glomerulus area but increased MMP-9 protein content in CTRL. The treatment with CAF (75 mg/kg) prevented the impairment of renal function, alteration of cortical Na^+^ transporters, and the fibrotic changes caused by the I/R process. In conclusion, while the data revealed potential side effects associated with the treatment, CAF proved effective in mitigating the alterations induced by the I/R process.

## Graphical abstract

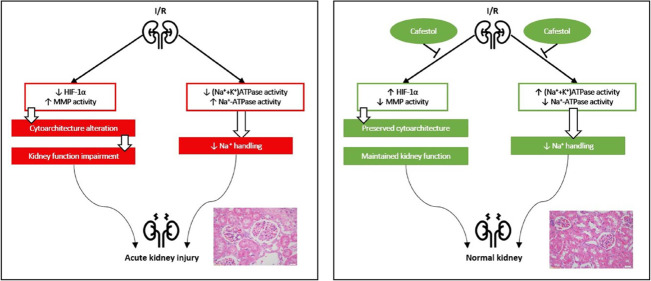

## Introduction

Acute kidney injury (AKI) is a prevalent and potentially life-threatening condition. Due to the difficulties in establishing criteria for AKI diagnostics, AKI is generally defined as an abrupt decrease in kidney function within hours, which encompasses both structural damage and loss of function.^1^ The syndrome manifests as a significant rise in serum creatinine, accumulation of nitrogen metabolism products, and decreased urine output, which are parameters for AKI classification.^2^ This condition is frequently observed in hospitalized patients (10–15%), and its prevalence can exceed 50% in intensive care units.^2^ In addition, the incidence of AKI in delayed graft function due to prolonged cold ischemia time is up to 50%. AKI is associated with significant morbidity, mortality, and healthcare costs.^3^ Depending on the severity of the insult, the renal damage can range from early stages of renal impairment to overt loss of kidney function requiring renal replacement therapy.^4^

Renal ischemia-reperfusion injury (IRI) is a key physiopathological mechanism underlying acute kidney injury (AKI).^1^ Brief periods of kidney ischemia disrupt metabolic processes, resulting in anaerobic metabolism, ATP depletion, and mitochondrial damage. The subsequent reperfusion phase exacerbates cellular injury through mechanisms that culminate in cell death.^5,6^ These disturbances in cellular homeostasis activate adaptive responses, including hypoxia-inducible factors (HIFs), adenosine monophosphate-activated protein kinase (AMPK), and heat shock proteins, which aim to mitigate stress-induced damage.^7^ However, several maladaptive signaling pathways are activated when kidney homeostasis fails to recover. We have previously demonstrated that 24 h after a renal bilateral I/R surgery, there is an increase in total metalloproteases metalloprotease activity and an imbalance between the two primary Na^+^ transporter activities.^8^ Altogether, these mechanisms may contribute to renal damage, fibrosis, and kidney function loss.

Cafestol (CAF) is a natural furanoditerpenoid found in the lipid fraction of coffee beans (Coffea arabica and C. canephora, commercial species). It predominantly exists as fatty esters, which are extracted into beverages mainly without the use of filters.^9^ It has been demonstrated that CAF exhibits a wide variety of pharmacological activities, including anti-inflammatory and antioxidative effects in different disease models.^10^ In an *in vitro* and *in vivo* model of cardiac fibrosis mediated by diabetes, CAF treatment showed cardioprotective effects through an antioxidant pathway.^11^ Moreover, in a doxorubicin-induced cardiotoxicity rat model, CAF reduced inflammatory mediators such as TNF-α and IL-1β and inhibited cardiac apoptosis.^12^ In the liver, CAF displayed a protective effect against hepatic IRI through antiapoptotic and antiautophagic mechanisms.^13^

Despite promising protective effects in the heart and liver, the effect of CAF on protecting renal function is unknown. Prospective analysis and randomized studies indicate the beneficial effect of coffee consumption on kidney function.^14,15^ Ren et al.^10^ associated a decrease in cardiovascular risk with active compounds caffeine, chlorogenic acid, kahweol, and CAF. Therefore, the present study aimed to investigate the pharmacological potential of CAF in renal IRI. We hypothesize that a single oral administration of CAF before the ischemic insult maintains renal cytoarchitecture and function in a well-established renal ischemic-reperfusion (I/R) rat model.

## Results

### Impact of CAF Pretreatment on Renal Function of CTRL and I/R Rats

Physiological parameters from CTRL and I/R rats pretreated or not with CAF are presented in Table 1. There was no difference between groups for the 24 h water intake. The CTRL rats pretreated with CAF showed decreased 24 h urine volume. In this condition, CAF_50_ and CAF_75_ reduced urine volume by 34 and 40%, respectively, compared to CTRL. I/R procedure did not alter 24 h urine volume but increased kidney index (18%). CAF treatment (CAF_50_ and CAF_75_) normalized the kidney index without changing the 24 h urine volume in the I/R rat.

**Table 1:** Impact of CAF Pretreatment on Physiological Parameters^a^

**Physiological Parameters**	**CTRL**	**CTRL+CAF**_**50**_	**CTRL+CAF**_**75**_	**I/R**	**I/R+CAF**_**50**_	**I/R+CAF**_**75**_
water intake (mL/24 h)	28 ± 1	24 ± 2	22 ± 1	26 ± 3	22 ± 0.3	29 ± 2
urine volume (mL/24 h)	15 ± 0.6	10 ± 1.4*	9 ± 0.3*	14 ± 0.8	11 ± 1.5	13 ± 1.2
kidney index (mg/g)	8.7 ± 0.2	8.7 ± 0.3	8.6 ± 0.2	10.3 ± 0.2*	8.4 ± 0.2**	8.9 ± 0.3**

a The data are presented as mean ± SEM. *p < 0.05 compared with CTRL; **p ≤ 0.0005 compared with I/R.

The impact of CAF pretreatment on kidney function is depicted in Table 2. As expected, there is a decrease in renal filtration of the I/R rats: (i) blood urea nitrogen (BUN; 140%) and plasma creatinine (PCre; 166%) accumulation; (ii) intense proteinuria (220%); (iii) reduced urinary creatinine (UCre; 45%); (iv) and reduced UCre/PCre ratio (55%). Only CAF_75_ prevented the reduction of all of the renal parameters evaluated in the I/R rats. CAF_50_, *per se*, increased both UCre and PCre, without changing the UCre/PCre ratio in CTRL rats.

**Table 2:** Impact of CAF Pretreatment on Renal Function^a^

**renal parameters**	**CTRL**	**CTLR+CAF**_**50**_	**CTRL+CAF**_**75**_	**I/R**	**I/R+CAF**_**50**_	**I/R+CAF**_**75**_
BUN (mg/dL)	53 ± 2.3	53 ± 4	52 ± 3.4	74 ± 3.3*	51 ± 4.1**	62 ± 3.4**
proteinuria (mg/dL)	39 ± 3.4	60 ± 13	37 ± 4.7	86 ± 3.8*	77 ± 7.8	51 ± 12**
UCre (mg/dL)	22 ± 1	45 ± 5.9*	20 ± 2.2	12 ± 1.8*	15 ± 2.4	22 ± 3.5**
PCre (mg/dL)	0.3 ± 0.02	0.5 ± 0.04*	0.4 ± 0.05	0.5 ± 0.05*	0.6 ± 0.04	0.3 ± 0.05**
UCre/PCre ratio	73 ± 6.7	88 ± 14.3	47 ± 6.5	33 ± 5.3*	27 ± 3.4	83 ± 7.8**

a Abbreviations: BUN, blood urea nitrogen; UCre, urinary creatinine; PCre: plasmatic creatinine. The data are presented as mean ± SEM. *p < 0.05 compared with CTRL. **p < 0.05 compared with I/R.

### Effect of CAF Pretreatment on Kidney Na^+^ Handling and Renal Cortical Primary Na^+^ Transporters

Plasma Na^+^ concentration (P_Na_) did not vary under the experimental conditions. The I/R procedure provoked a reduction of filtered load Na^+^ (FL_Na_) (66%) when compared to CTRL rats (Table 3). Consequently, there was a decrease in urinary Na^+^ excretion (U_Na_V) (47%), also reflected by the decline of fractional Na^+^ excretion (FE_Na_) (40%) (Table 3). CAF_75_ pretreatment exclusively prevented the reduction in FL_Na_, as U_Na_V and FE_Na_ reached levels even lower than those observed in the I/R rats. CAF_75_ administered to CTRL rats provoked a ∼ 40% decline in renal function.

**Table 3:** Impact of CAF Pretreatment on Na^+^ Handling^a^

**Na** ^**+**^ **parameters**	**CTRL**	**CTRL+CAF**_**75**_	**I/R**	**I/R+CAF**_**75**_
P_Na_ (mEq/L)	203 ± 11	222 ± 11	215 ± 14	215 ± 15
U_Na_V (mEq/100g body weight)	1.0 ± 0.08	0.4 ± 0.08*	0.5 ± 0.1*	0.3 ± 0.04
FL_Na_ (mEq/min)	0.18 ± 0.02	0.11 ± 0.01*	0.06 ± 0.01*	0.14 ± 0.02**
FE_Na_ (%)	0.68 ± 0.07	0.44 ± 0.06*	0.41 ± 0.08*	0.19 ± 0.02

a Abbreviations: P_Na_: plasma Na^+^ concentration; U_Na_V: urinary Na^+^ excretion; FL_Na_: filtered load Na^+^; and FE_Na_: fractional excretion of Na^+^ (the percentage of FL_Na_ that was excreted in the urine). The data are presented as mean ± SEM. *p < 0.05 compared with CTRL; **p < 0.05 compared with I/R.

We also measured the activity of the primary renal cortical Na^+^ transporters: (Na^+^+K^+^)-ATPase and Na^+^-ATPase. The (Na^+^+K^+^)-ATPase activity was 31% lower in the I/R (Figure 1a), whereas the Na^+^-ATPase activity was ∼ 3 times higher (Figure 1b). CAF_75_ pretreatment prevented the imbalance between the ATPases. CAF_75_ administered to CTRL rats did not affect the enzymes activities. The protein content of (Na^+^+K^+^)-ATPase was not altered in any experimental groups (Figure 1c).

**Figure 1:**
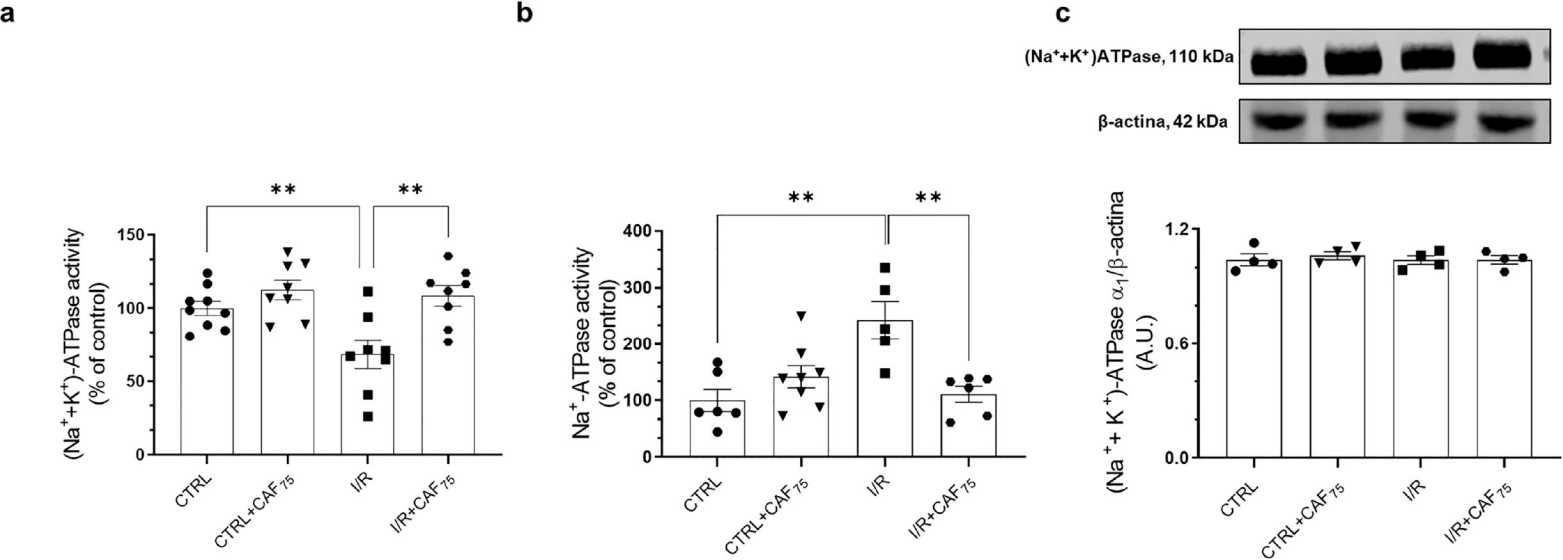
CAF pretreatment prevented IRI-induced alterations on the renal cortex primary Na^+^ transporters. (A) Ouabain-sensitive (Na^+^+K^+^)-ATPase activity. (B) Ouabain-resistant, furosemide-sensitive Na^+^-ATPase activity. (C) (Na^+^+K^+^)­ATPase protein content. Upper panel: representative images of (Na^+^+K^+^)­ATPase and β-actin detections. Lower panel: densitometric analysis of the immunoreactive band ratio between (Na^+^+K^+^)-ATPase and β-actin detections. The data are presented as the mean ± SEM **p < 0.01.

### Effect of CAF Pretreatment on Tissue Injury Promoted by Renal I/R

Figures 2 and 3 show representative cortical and medullary images of hematoxylin–eosin staining in all experimental groups. A histological image of a kidney from a typical rat (with no vehicle administration nor submitted to a surgery process) is shown in the Supporting Information for reference (Figure S1). The kidneys of CTRL rats presented normal morphology, with distinguishable characteristics of proximal and distal tubules. The glomerulus presented a typical appearance (Figure 2a,e). The tissue injury was characterized in the I/R rat cortex by (i) space between the tubules (asterisk), (ii) dilation of tubules (arrow), (iii) cast protein inside the tubules (black circle), (iv) glomerular segmentation (star), and (v) detached necrotic tubular cells (hollow black circle) (Figure 2c,g). Moreover, the glomerulus area was decreased (21%, Figure 2i). However, the Bowman’s space did not change (Figure 2j). In the medulla of the I/R rats, we detected deposition of amorphous material in the lumen of the tubule (closed circle) and the presence of inflammatory infiltrate (closed square) (Figure 3c,g). According to morphological observation, CAF_75_ pretreatment prevented kidney injury in both cortex and medulla of the I/R rat (Figures 2d,h and 3d,h) but did not block the shrinkage in glomerulus area (Figure 2i). Although the kidney cytoarchitecture of CTRL and CTRL+CAF_75_ (Figures 2b,f and 3b,f) was similar, we detected a 19% decrease in the glomerulus area of the CTRL+CAF_75_ in comparison to CTRL (Figure 2i).

**Figure 2:**
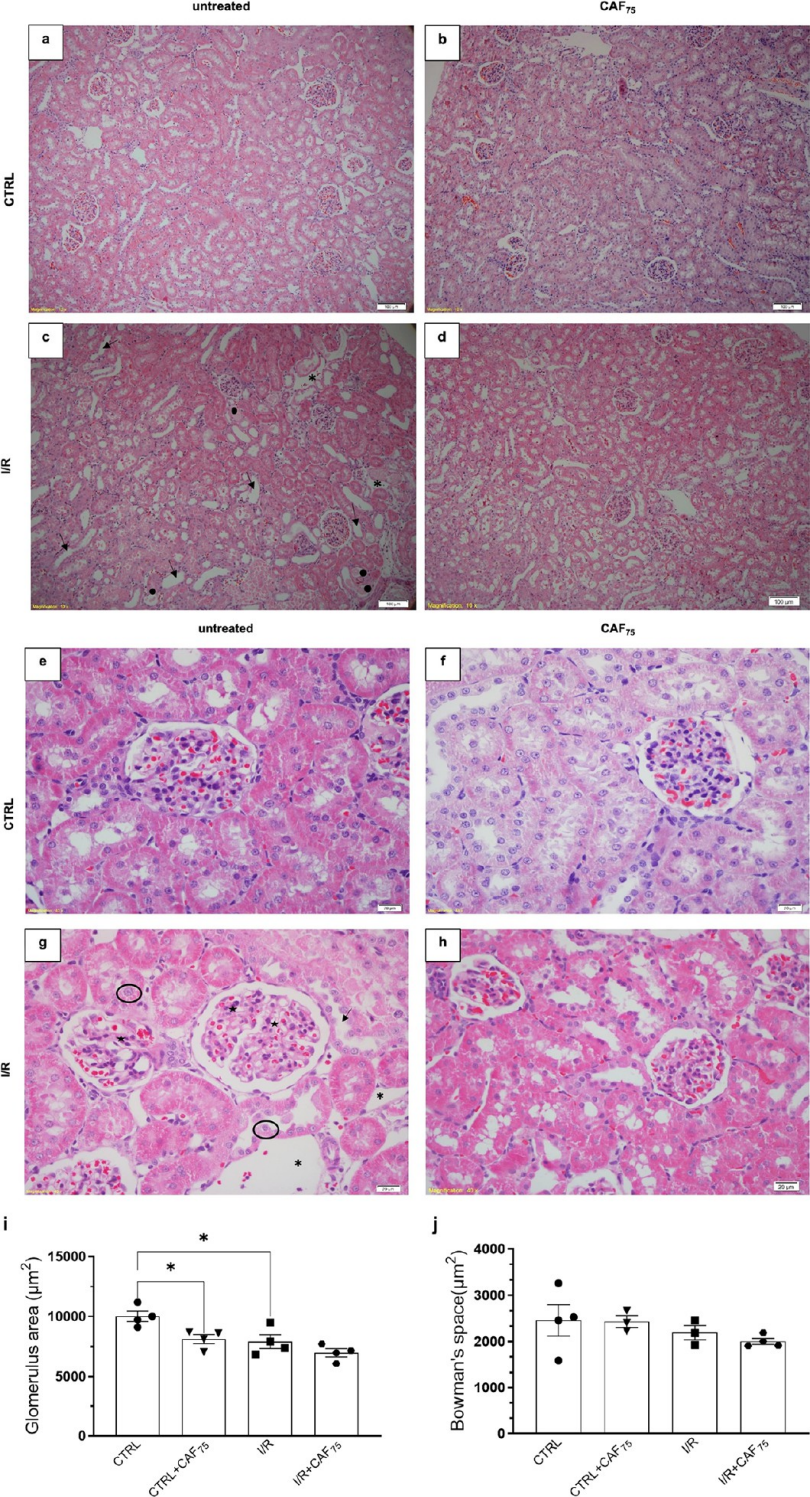
CAF pretreatment partially prevented cortical kidney damage induced by renal I/R. Kidney damage is demonstrated: asterisk: space between the tubules, arrow: dilation of tubules, black circle: cast protein inside the tubules, star: glomerular segmentation, hollow black circle: detached necrotic tubular cells. Representative photomicrographs (a–d: 100× and e–h: 400× magnifications) of cortical kidney sections (3 μm) stained with hematoxylin-eosin from CTRL (a, e); CTRL+CAF_75_ (b, f); I/R (c, g); I/R+CAF_75_ (d, h). For each group, *n* = 4. (i) Glomerulus area; (j) Bowman’s space. The data are presented as the mean ± SEM *p < 0.05.

**Figure 3:**
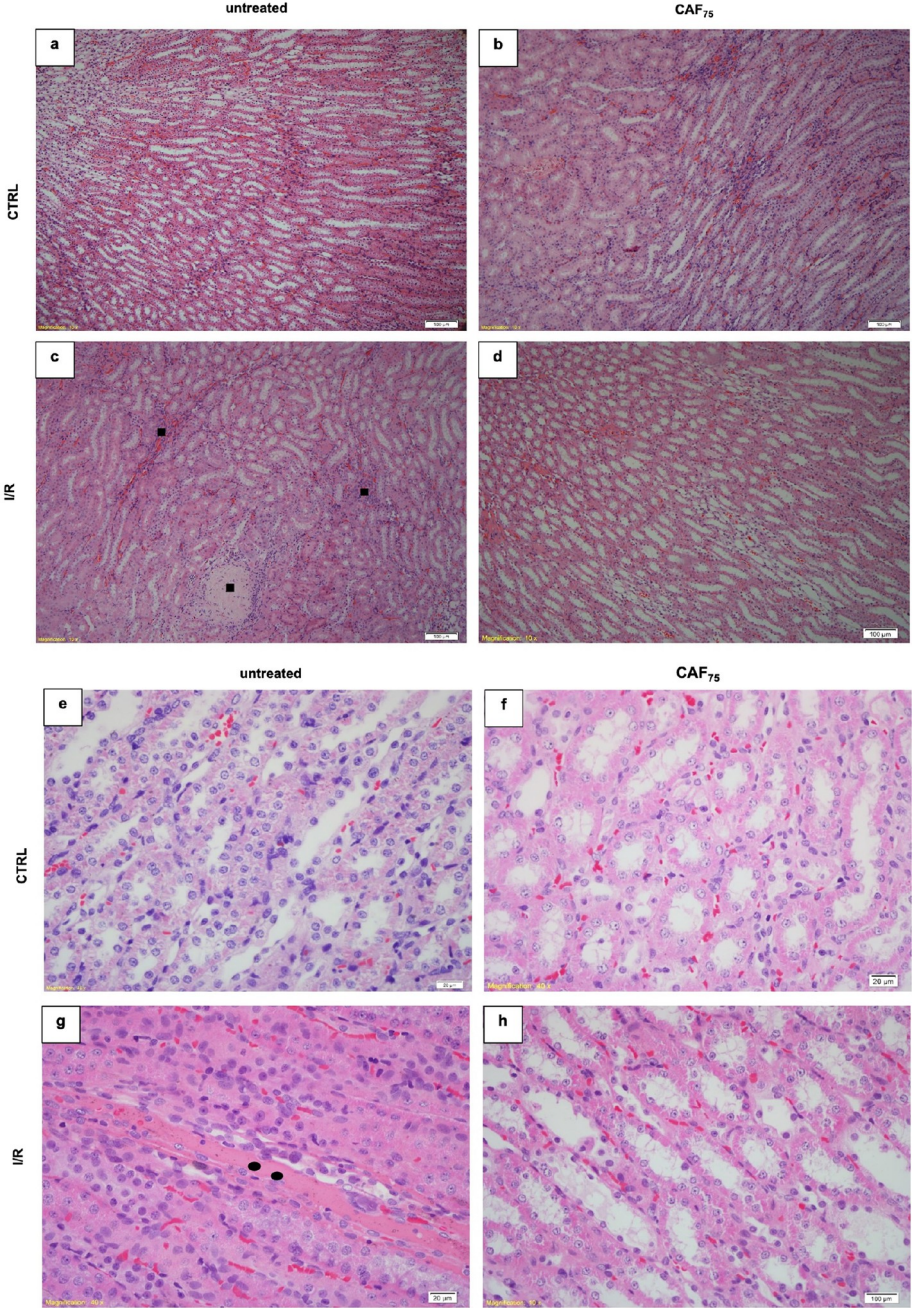
CAF pretreatment partially prevented medullary kidney damage induced by renal I/R. Kidney damage is demonstrated: black square: inflammatory infiltrate; closed circle: deposition of amorphous material in the lumen of the tubule. Representative photomicrographs (a–d: 100× and e–h: 400× magnifications) of medullary kidney sections (3 μm) stained with hematoxylin-eosin from CTRL (a, e); CTRL+CAF_75_ (b, f); I/R (c, g); I/R+CAF_75_ (d, h). For each group, *n* = 4.

Extracellular matrix collagen was detected in the renal parenchyma with Picrosirius red staining (Figure 4). I/R rats presented 2 times collagen accumulation, mainly around the medullary tubules (please compare Figure 4c,g with Figure 4a,e, respectively; for quantification, see Figure 4i,j). The pretreatment with CAF_75_ attenuated the interstitial collagen deposition (Figure 4d,h–j). CAF_75_, *per se*, did not promote collagen deposition in CTRL rats (Figure 4i,j).

**Figure 4:**
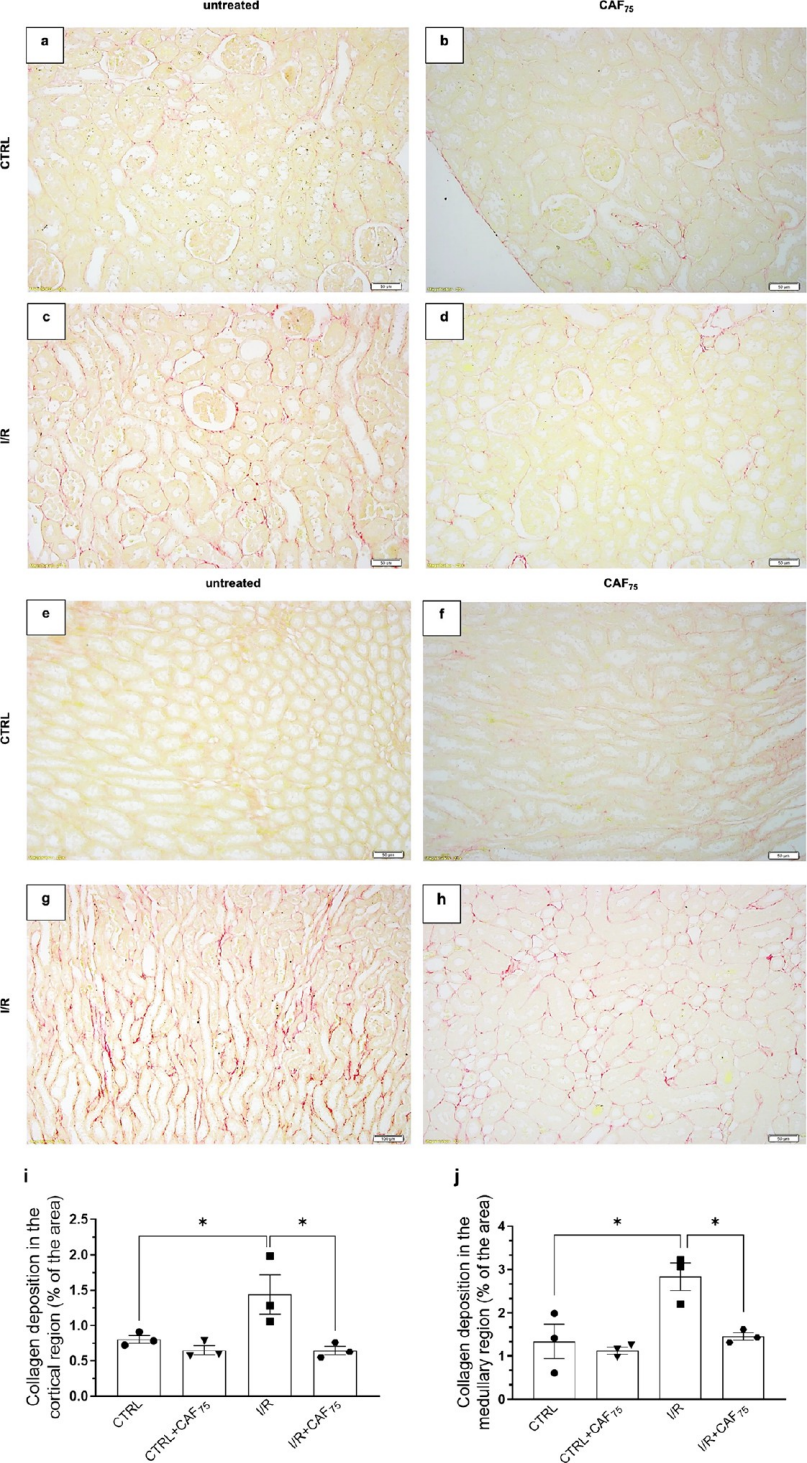
CAF pretreatment prevented collagen deposition in the I/R rat kidney. Representative photomicrographs (a–d: cortex; e–h: medulla) of kidney sections (3 μm) stained with Picrosirius red. CTRL (a, e); CTRL+CAF_75_ (b, f); I/R (c, g); I/R+CAF_75_ (d, h). For each group, *n* = 3. Quantification of collagen deposition in the cortical (i) and medullary region (j). The data are presented as the mean ± SEM *p < 0.05.

### Molecular Mechanism Involving CAF Nephroprotection in I/R-Induced AKI

To investigate the molecular mechanism underlying CAF nephroprotection, we determined the HIF-1α protein content and matrix metalloprotein-9 (MMP-9) expression and activity (Figure 5). Kidney I/R provoked in the kidney cortex: (i) 52% reduction in HIF-1α (Figure 5a) and (ii) 43% increase in MMP-9 protein content and activity (Figure 5b–5d). The densitometry of the uncolored bands shows that I/R rats presented a 357% increase in MMP-9 activity compared with the CTRL group (Figure 5d). CAF_75_ prevented the reduction of HIF-1α, but not the MMP-9 protein content (Figure 5a,b) in the I/R rat. Additionally, CAF_75_ partially prevented the increase in the MMP-9 activity (Figure 5d). In the CTRL rats, CAF_75_ provoked an increase in 43% in the MMP-9 protein content without changing its activity (Figure 5b,d).

**Figure 5:**
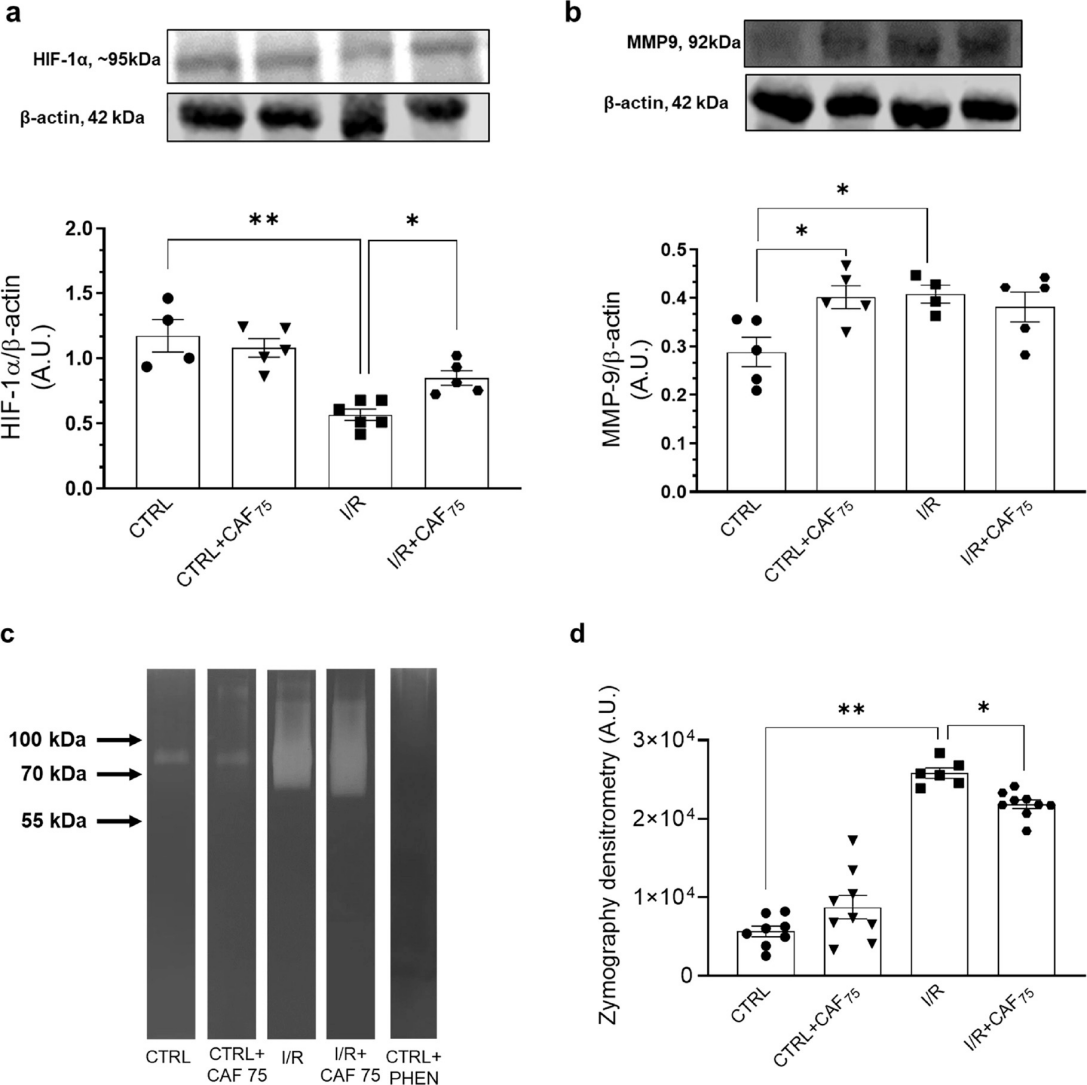
CAF pretreatment prevented kidney fibrosis markers on the renal cortex. (a) HIF-1α protein content. Upper panel: representative images of HIF-1α and β-actin detections. Lower panel: densitometric analysis of the immunoreactive band ratio between HIF-1α and β-actin detections. (b) Matrix metalloproteinase 9 (MMP-9) protein content. Upper panel: representative images of MMP-9 and β-actin detections. Lower panel: densitometric analysis of the immunoreactive band ratio between MMP-9 and β-actin detections. (c) Representative images of MMP activity by zymography. Phenanthroline (PHEN) is a potent inhibitor of zinc metalloproteinase. (d) Quantification of the MMP activity by densitometry. The data are presented as the mean ± SEM *p < 0.05; ** *p* < 0.01.

## Discussion

We provide evidence that oral administration of 75 mg/kg of CAF, 2 h before the I/R procedure, prevents kidney filtration function decay, the impairment of renal Na^+^ handling, and the disruption of renal cytoarchitecture and collagen deposition. Additionally, we proposed that CAF nephroprotection involves, at least in part, the maintenance of cortical HIF-1α levels and zinc-dependent MMP. Both mechanisms are associated with decreased collagen deposition and kidney structure and function preservation.

Most studies examine Arabica coffee as a beverage, rather than its constituent molecules. Moderate coffee consumption has been associated with a lower risk of chronic kidney disease (CKD).^16^ A prospective study analyzed the correlation between habitual coffee consumption, estimated glomerular filtration rate (eGFR), and the urine albumin-creatinine ratio in patients from a Dutch city.^15^ There was no evidence of an association between coffee consumption and eGFR or the albumin-creatinine ratio longitudinally during follow-up. However, the results suggested that higher coffee consumption may help preserve eGFR among high-risk groups for CKD, such as patients over 70 years old and obese patients.^15^ Another study used Mendelian randomization, analyzing genetic variations that affect exposure to modifiable risk factors to estimate a causal association between exposure and outcomes. The analyses showed that increased coffee consumption offers protection against CKD and is associated with increased eGFR.^14^

The green and roasted Arabica coffee bean contains several known bioactive substances, such as caffeine, chlorogenic acids, and diterpenes.^17^ Among the known diterpenes, CAF and kahweol are the main substances. They are present in the lipid fraction of coffee and are used to identify coffee adulterations due to their high chemical stability.^18^ These diterpenes are pentacyclic alcohols containing a furan ring, differing only by an unsaturation at C1–C2 (in kahweol). The diterpenes are primarily found in their esterified form with fatty acids (98%), while their free form is minor (2%).^19^ Both compounds are characterized by low stability when exposed to heat, acids, and light, with kahweol being particularly unstable in its free form. Due to the minimal differences in their molecular structures, separating these compounds through chromatographic techniques is challenging.^18^ Lima et al.^20^ reported a fast and highly selective procedure for hydrogenating a mixture of CAF and kahweol to obtain pure CAF. Alone, CAF is bioaccessible, is available for absorption in its alcohol form, and suffers an oxidation reaction during the oral phase of digestion.^21^

As reported previously, we reproduced the renal I/R model with moderate tissue injury, decreased kidney function, and disruption in tubular Na^+^ handling.^8,22^ This model mimics ischemic AKI in humans.^23^ Male rats were selected to minimize potential variability due to hormonal fluctuations in females, which could have introduced additional complexity to the data interpretation. This approach was intended to maintain a controlled preclinical model and enhance the reproducibility of our findings. However, we recognize the importance of studying both sexes and acknowledge that future research should include female rats to assess potential sex-based differences in the observed effects.

We analyzed two doses of CAF, 50 and 75 mg/kg, to prevent such alterations. Albeit the lowest dose protected against the increase in kidney index and BUN, it did not prevent proteinuria, the reduction of UCre, and the increase in PCre. In this way, we chose a single dose of 75 mg/kg of CAF administered 2 h before I/R as the nephroprotective condition. The nephroprotection is desirable in controlled clinical interventions that could lead to the I/R process, such as cardiac surgery or kidney transplantation.^24^

CAF, a bioactive molecule derived from coffee, is known for its hypercholesterolemic effects in individuals who consume excessive amounts of boiled coffee, a nonfiltered hot beverage.^9^ Despite this, CAF has shown therapeutic potential in various injury models.^10^ In cardiac studies, doses ranging from 1 to 5 mg/kg have been reported to reduce oxidative stress and promote cardioprotection in different models of heart disease.^11,12^ Similarly, in hepatic models, CAF pretreatment for 3 to 5 days at doses between 10 and 100 mg/kg prevented increases in hepatic enzymatic markers, mitigated oxidative stress, and reduced tissue damage.^13,25^ A common feature among these studies is administering low to moderate doses of CAF over multiple consecutive days. To replicate this effect, we selected intermediate doses and administered them as a single dose 2 h before the procedure. Our findings demonstrated a nephroprotective effect at 75 mg/kg of CAF. Importantly, both doses (50 and 75 mg/kg) were well tolerated and did not induce toxicity in Wistar rats, as confirmed by ref 26, although we observed some minor renal alterations.

Pharmacokinetics data demonstrated that CAF is metabolized after 24 h postgavage administration.^27^ CAF is concentrated in the gastrointestinal tract of mice, mainly in the small intestine and liver. It was reported that less than 1% is detected in the kidney after 2 h of administration of 1.5 mg CAF. This is in accordance with other reports.^28,29^ The kidneys are the main elimination routes of xenobiotics. Due to the elimination function, the kidneys could be more susceptible to CAF or its metabolites than other tissues, especially in higher doses, as used in our work. Based on these findings, CAF metabolites can also be responsible for nephroprotection during I/R or for the specific renal changes provoked by CAF in healthy kidney rats.

In healthy rats, CAF-induced glomerulus area reduction is associated with a decrease in FL_Na_, which may explain the decline in urine volume. This data opposes the observation in refs 11,12 since CAF did not change in tissue morphology. We also detected an increase in MMP-9 protein content in the cortical kidney of CTRL+CAF_75_ rats, which was not accompanied by the augmentation of its activity. Based on these results, we hypothesize that the increased MMP-9 protein levels in the glomerulus may disrupt the pathways involved in the filtration process, reducing the glomerular area and potentially contributing to decreased FL_Na_ levels. Consequently, the reduction in FL_Na_ may have resulted in decreased urine volume, as no changes were observed in the primary renal cortical Na^+^ transporters.

To minimize the potential deleterious effects of CAF in healthy animals, future studies could explore lower doses (ranging from 10 to 75 mg/kg) administered over 3 to 5 consecutive days before establishing the AKI model. Repeated administration may better simulate a preconditioning effect, reducing acute kidney alterations while preserving renal protection. Since previous studies have not reported deleterious effects at lower doses in other organs,^10–13,25^ assessing renal function under these conditions will be essential to determine the nephroprotective potential of CAF against AKI.

The kidney cortical tubules are responsible for 70% of Na^+^ reabsorption from the ultrafiltrate, and any changes in Na^+^ reabsorption can influence the final urinary composition and volume.^30^ (Na^+^+K^+^)­ATPase is the main sodium pump responsible for Na^+^ reabsorption.^31,32^ I/R rats presented a disruption in tubular Na^+^ handling, as seen by the decrease in the levels of U_Na_V and FE_Na_. Despite that, ouabain-sensitive (Na^+^+K^+^)­ATPase activity was reduced in this group. I/R provoked a decrease in intracellular ATP levels, inhibiting the (Na^+^+K^+^)­ATPase activity, as seen by others.^5,33–35^ The second Na^+^ pump present in the proximal tubules – ouabain-resistant, furosemide-sensitive Na^+^-ATPase – allows fine adjustment in U_Na_V because of its low capacity of transport but high Na+ affinity.^36^ The augmentation of Na^+^-ATPase activity may be an adaptive response to the low activity of (Na^+^+K^+^)­ATPase, increasing Na^+^ reabsorption in response to I/R. CAF pretreatment maintains both cortical ATPase activities close to CTRL levels but could not prevent disturbances in renal Na^+^ handling. We cannot rule out the contribution of tubular-glomerular balance and the activity of Na^+^ transporters in the medulla, which CAF pretreatment may not prevent.

Fibrosis is pointed out to trigger renal I/R injury and is the main mechanism associated with the development of chronic kidney disease (CKD).^37^ We have previously demonstrated that immunostaining of renal tissue derived from the I/R model is positive for TGF-β1 and fibronectin and associated with an augmented collagen deposit.^8,22^ Cortes et al.^8^ also demonstrated the augmentation of MMP-9 activity and protein content in the renal cortex of I/R rats, which is similar to our finding. An increase in MMP-9 activity on the glomerulus of the renal I/R model was associated with the degradation of zonula occludens-1 (ZO-1) protein, a molecular component of tight junctions.^32^ This mechanism can be associated with a greater collagen deposition around the gloms, which may reduce the filtration rate and FL_Na_ of the I/R rats. The increased MMP-9 activity may also affect Na^+^ transporters and, ultimately, Na^+^ handling.^38,39^

Furthermore, there is increasing evidence of the pathophysiological role of MMP-9 in establishing fibrosis during the AKI and in the transition to CKD.^40,41^ We showed that CAF treatment partially prevented the increase in MMP-9 activity induced by the I/R. Liu et al.^11^ described a similar result in a model of diabetes-induced cardiac fibrosis. It was shown that CAF presented an antifibrotic action by regulating antioxidant genes that repressed collagen signaling under a hyperglycemic state.^11^

We have also demonstrated that HIF-1α is reduced in the cortical region of I/R rats. Emerging evidence demonstrated the dual roles of HIFs in renal repair after AKI.^42^ Adaptive repair after mild AKI is associated with the rapid and transient activation of HIF, whereas maladaptive repair following severe AKI is associated with a delayed and sustained HIF response. In our model, the decreased levels of HIF-1α after 24 h from the I/R procedure suggest that maladaptive repair is the main profile. In the kidney, reduced HIF-1α activity is related to increased fibrosis and damage.^11,43,44^ In I/R models, pretreatment with pharmacological inhibitors of prolyl hydroxylases (PHD), which activate HIFs, significantly reduced ischemic renal injury, reduced apoptosis, and upregulated HIF target genes.^45^ Moreover, Jamadarkhana et al.^46^ found that administering the PHD inhibitor before and after the onset of renal bilateral ischemia activated HIF-1α expression and attenuated renal parameter impairment. Our data are in accordance with the literature since CAF pretreatment prevented the reduction of HIF-1α induced by I/R, culminating in decreased collagen deposition. We conclude that CAF is a nefroprotective substance by blocking HIF-1α and MMP-9 activation.

## Conclusions

Our study demonstrates that oral administration of 75 mg/kg CAF preserved kidney function, renal cortical primary Na^+^ transporter activity, and tissue cytoarchitecture following renal I/R. CAF partially attenuated MMP-9 activity and maintained HIF-1α protein levels, thereby preventing collagen deposition. Despite these benefits, CAF also altered renal parameters in healthy rats, indicating potential side effects. These findings highlight the CAF nephroprotective mechanism and its potential as a therapeutic intervention for ischemic renal injury.

## Methods

### CAF Extraction

CAF isolation was performed from raw coffee beans (Coffea arabica L.) according to the protocol developed by ref 47. The identity of the molecule was confirmed by high-resolution mass spectrometry (HRMS), ^1^H and ^13^C nuclear magnetic resonance (NMR), and its melting point. CAF was obtained up to 98% purity (see the Supporting Information).

### Experimental Design

Fifty-seven male Wistar rats (180–220 g) were purchased from “*Biotério Central de Ratos*”, located at Federal University of Rio de Janeiro (UFRJ), Brazil. All procedures involving rats were carried out in accordance with the rules of Good Practices and approved by the Ethics Committee for the Use of Animals (CEUA) of the Federal University of Rio de Janeiro (UFRJ) under the number 073/21. The rats were kept during the study period in an appropriate *vivarium* with constant temperature (23 ± 3 °C) in the standard light/dark cycle (12/12 h).

The rats were randomly divided into two large groups: (i) CTRL groups: submitted to sham surgery; and (ii) I/R groups: submitted to bilateral ischemia for 30 min followed by 24 h reperfusion. Rats were anesthetized with ketamine (50 mg/kg, intraperitoneal, Crystalia, São Paulo, Brazil) and xylazine (5 mg/kg, intraperitoneal, Syntec, São Paulo, Brazil). Renal I/R was induced under aseptic conditions by applying a nontraumatic vascular clamp in both renal pedicles for 30 min.^8^ Both CTRL (*n* = 14), and I/R (*n* = 18), groups were separated in CAF-pretreated groups, which were administrated in doses of 50 and 75 mg/kg (via oral) by gavage 2 h before ischemia, resulting in 4 groups: CTRL+CAF_50_ (*n* = 4), CTRL+CAF_75_ (*n* = 10), I/R+CAF_50_ (*n* = 8), and I/R+CAF_75_ (*n* = 10). During the 24 h reperfusion period, rats were individually housed in metabolic cages for 24 h of urine and 24 h of water intake measurements. Afterward, rats were euthanized, and blood and kidneys were harvested. We thoroughly analyzed all physiological and renal parameters from typical rats (pure control group), and the results were statistically comparable to those of the untreated sham control group (data not shown). Thus, the presented data represent the untreated sham control group as CTRL in the analysis.

### Urine and Blood Analysis

The blood samples were collected after euthanasia in glass tubes containing 5 mM EDTA and centrifuged at 3000 *g* for 10 min for plasma fraction separation to measure blood urea nitrogen (BUN), Na^+^, and creatinine. Urine samples were centrifuged for 5 min to eliminate the sediments prior to proteinuria, Na^+^, and creatinine analysis. Urinary (UCre) and plasma creatinine (PCre), BUN, and proteinuria were measured by spectrophotometry using specific colorimetric kits (Gold Analisa Diagnóstica Ltd., Belo Horizonte, Brazil). The renal function parameters were calculated as previously described.^8^ The concentration of Na^+^ in urine and blood was measured by flame spectrometry (Analyzer 910 MS, Analyzer, São Paulo, Brazil).

### Kidney Cortex Homogenate and (Na^+^+K^+^)­ATPase and Na^+^-ATPase Activities

Kidney cortex homogenate preparation from the kidney cortex was obtained as described previously.^36^ Total protein concentration was assayed according to Lowry et al.,^48^ using bovine serum albumin (BSA) as a standard. Samples were maintained at – 20 °C until use. The (Na^+^+K^+^)-ATPase and Na^+^-ATPase activities were measured as previously described.^49^

### Renal Histology

Parafin-embedded left kidney sections (3 μm) were stained with hematoxylin-eosin (HE) to evaluate renal morphology and Picrosirius red to detect collagen deposition.^8,50^ Histological analysis was performed using a microscope (DP72 Microscope Digital Camera attached to an Olympus BX53F microscope, Japan). The glomeruli morphometric analysis was assessed as described by ref 51, and the quantification of collagen deposits was measured as described by ref 52 using the ImageJ software (National Institutes of Health, Bethesda, MD).

### Western Blot Analysis

One hundred and 20 micrograms of protein from kidney cortex homogenates were separated by electrophoresis in a polyacrylamide gel electrophoresis (10% SDS-PAGE) and transferred to nitrocellulose membranes (10600003; GE Healthcare Life Sciences, Freiburg, Germany). After blocking (1 h with 5% milk), the membranes were incubated with specific primary antibodies: anti-(Na^+^+K^+^)-ATPase (1:1000, A276, Sigma-Aldrich, Saint Louis, MO), anti-MMP-9 (1:1000, 2270, Cell Signaling Technology, Danvers, MA), and anti-HIF-1α (1:500, SAB5200017, Sigma-Aldrich, Saint Louis, MO) for 16 h at 4 °C. The membranes were successively washed with TBS-T buffer solution, followed by incubation with fluorescence-conjugated secondary antibody, at a concentration 10 times more diluted than the primary antibody used (antimouse IRDye 680RD and antirabbit IRDye 800CW, Li-Cor). The immunofluorescence was detected using the Odyssey System (Li-Cor Bioscience, Lincoln, NE) for infrared imaging recording, and band intensities were quantified using ImageJ software (National Institutes of Health, Bethesda, MD). Blots were stripped and reprobed using β-actin monoclonal antibody (1:1000; A5316, Sigma-Aldrich, Saint Louis, MO) as a loading control.

### Zimography

Proteolytic activities of matrix metalloproteinases (MMPs) were assayed using 7.5% SDS-PAGE containing 0.1% gelatin (G2500; Sigma-Aldrich, Saint Louis, MO) incorporated into the gel as the proteinaceous substrate.^53^ Twenty-five micrograms of protein from cortex homogenates were diluted in a sample buffer (125 mM Tris, pH 6.8, 4% SDS, 20% glycerol, and 0.002% bromophenol blue). Electrophoresis was performed in accordance to ref 8. Then, SDS was removed by incubation of the gels with 2.5% Triton X-100 at 4 °C. The gels were incubated for 48 h at 37 °C in 10 mM glycine–NaOH buffer, pH 10, in either the absence or presence of 10 mM 1,10-phenanthroline (PHEN), a potent zinc metallopeptidase inhibitor (P9375; Sigma-Aldrich, Saint Louis, MO). The gels were stained with 0.2% Coomassie Brilliant Blue R-250 in methanol-acetic acid–water (50:10:40) and destained with a methanol-acetic acid–water (5:10:85) solution. The molecular mass of sample polypeptides was calculated from the mobility of molecular mass standards (Thermo Fisher Scientific, MA, USA). The gels were photographed and quantified by the IMAGE J program (NIH, MD, USA).

### Statistical Analysis

The data are presented as the mean ± SEM. A one-way ANOVA followed by Sidak’s post hoc test was applied to detect differences of: (1) CAF in healthy rats: CTRL vs CTRL+CAF_50_ or CTRL+CAF_75_ rat; (2) I/R surgery: CTRL vs I/R, and (3) CAF treatment before I/R surgery: I/R vs I/R+CAF_50_ or I/R+CAF_75_. Statistical tests and graphs were generated using GraphPad Prism 8.0.2 software (GraphPad Inc., La Jolla, CA).

## Supplementary Material



## Data Availability

The data underlying this study are not publicly available due to the large volume and complexity of the raw data, which can not be made available in a publicly accessible format. The data are available from the corresponding author upon reasonable request.

## Notes

Additional methods details: purity of cafestol extraction and (Figure S1) histological image of a kidney from a typical rat (PDF)

## Abbreviations

I/R: ischemia-reperfusion
AKI: acute kidney injury
CAF: cafestol
IRI: ischemia-reperfusion injury
HIFs: hypoxia-induced factors
BUN: blood urea nitrogen
MMP: matrix metalloproteinase
PHEN: 1,10-phenanthroline
UCre: urinary creatinine
PCre: plasma creatinine
CKD: chronic kidney disease

## References

[1] Makris K., Spanou L. (2016). Acute Kidney Injury: Definition, Pathophysiology and
Clinical Phenotypes. Clin. Biochem. Rev..

[2] Ronco C., Bellomo R., Kellum J. A. (2019). Acute kidney
injury. Lancet.

[3] Sabet
Sarvestani F., Afshari A., Azarpira N. (2024). The role of non-protein-coding
RNAs in ischemic acute kidney injury. Front.
Immunol..

[4] Ostermann M., Cerdá J. (2018). The Burden
of Acute Kidney Injury and Related Financial
Issues. Contrib Nephrol.

[5] Chatauret N., Badet L., Barrou B., Hauet T. (2014). Ischemia-reperfusion:
From cell biology to acute kidney injury. Prog.
Urol.

[6] No̷rgård M. Ø., Svenningsen P. (2023). Acute Kidney Injury by Ischemia/Reperfusion and Extracellular
Vesicles. Int. J. Mol. Sci..

[7] Gallazzini M., Pallet N. (2018). Endoplasmic reticulum stress and kidney dysfunction. Biol. Cell.

[8] Cortes A. L., Gonsalez S. R., Rioja L. S., Oliveira S. S. C., Santos A. L. S., Prieto M. C., Melo P. A., Lara L. S. (2018). Protective outcomes
of low-dose doxycycline on renal function of Wistar rats subjected
to acute ischemia/reperfusion injury. Biochim.
Biophys. Acta, Mol. Basis Dis..

[9] Silva M. A. E., Brand A. L. M., Novaes F. J. M., Rezende C. M. (2022). Cafestol, Kahweol
and Their Acylated Derivatives: Antitumor Potential, Pharmacokinetics,
and Chemopreventive Profile. Food Rev. Int..

[10] Ren Y., Wang C., Xu J., Wang S. (2019). Cafestol and Kahweol:
A Review on Their Bioactivities and Pharmacological Properties. Int. J. Mol. Sci..

[11] Liu J.-C., Chen P.-Y., Hao W.-R., Liu Y.-C., Lyu P.-C., Hong H.-J. (2020). Cafestol
Inhibits High-Glucose-Induced Cardiac Fibrosis
in Cardiac Fibroblasts and Type 1-Like Diabetic Rats. Evidence-Based Complement. Altern. Med..

[12] Al-Kenany S. A., Al-Shawi N. N. (2023). Protective effect of cafestol against doxorubicin-induced
cardiotoxicity in rats by activating the Nrf2 pathway. Front. Pharmacol..

[13] Ji J., Wu L., Feng J., Mo W., Wu J., Yu Q., Li S., Zhang J., Dai W., Xu X., Mao Y., Xu S., Chen K., Li J., Guo C. (2020). Cafestol preconditioning
attenuates apoptosis and autophagy during hepatic ischemia-reperfusion
injury by inhibiting ERK/PPARγ pathway. Int. Immunopharmacol.

[14] Kennedy O. J., Pirastu N., Poole R., Fallowfield J. A., Hayes P. C., Grzeszkowiak E. J., Taal M. W., Wilson J. F., Parkes J., Roderick P. J. (2020). Coffee Consumption and Kidney Function:
A Mendelian Randomization Study. Am. J. Kidney
Dis.

[15] van
Westing A. C., Ochoa-Rosales C., van der Burgh A. C., Chaker L., Geleijnse J. M., Hoorn E. J., Voortman T. (2023). Association
of habitual coffee consumption and kidney function: A prospective
analysis in the Rotterdam Study. Clin Nutr.

[16] He W. J., Chen J., Razavi A. C., Hu E. A., Grams M. E., Yu B., Parikh C. R., Boerwinkle E., Bazzano L., Qi L., Kelly T. N., Coresh J., Rebholz C. M. (2021). Metabolites Associated
with Coffee Consumption and Incident Chronic Kidney Disease. Clinical Journal of the American Society of Nephrology.

[17] Jeszka-Skowron M., Zgoła-Grześkowiak A., Grześkowiak T. (2014). Analytical
methods applied for the characterization and the determination of
bioactive compounds in coffee. Eur. Food Res.
Technol..

[18] Benassi, M. D. T. ; Dias, R. C. E. Assay of Kahweol and Cafestol in Coffee, In Coffee in Health and Disease Prevention ( Preedy, V. R. Ed.), 2015, pp 993–1004. Elsevier BV.

[19] Tsukui A., Oigman S. S., Rezende C. M. (2014). Óleo
de Grãos de Café
Cru: Diterpenos Cafestol e Caveol. Revista Virtual
De Química.

[20] Lima F. A., Bezerra M. A. M., Souza R., Itabaiana I., Haynes T., Hermans S., Wojcieszak R., Novaes F. J. M., Rezende C. M. (2020). Fast and Highly Selective Continuous-Flow
Catalytic Hydrogenation of a Cafestol–Kahweol Mixture Obtained
from Green Coffee Beans. ACS Omega.

[21] Brand A., Silva A., Andriolo C., Mellinger C., Uekane T., Garrett R., Rezende C. (2024). Bioaccessibility of
Cafestol from Coffee Brew: A Metabolic Study Employing an *In Vitro* Digestion Model and LC-HRMS. J. Agric. Food Chem..

[22] Gonsalez S. R., Cortes A. L., Romanelli M. A., Mattos-Silva P., Curnow A. C., Prieto M. C., Einicker-Lamas M., Lara L. S. (2020). Lysophosphatidic Acid Prevents Ischemia Reperfusion
Injury but does not Prevent Tubular Dysfunction. J. Nephrol Sci..

[23] Bao Y.-W., Yuan Y., Chen J.-H., Lin W.-Q. (2018). Kidney disease models:
tools to identify mechanisms and potential therapeutic targets. Zool. Res..

[24] Ali A., Sampaio T. L., Khan H., Jeandet P., Akkol E. K., Bahadar H., Costa Martins A. M. (2022). Plants
with Therapeutic Potential
for Ischemic Acute Kidney Injury: A Systematic Review. Evidence-Based Complementary Altern. Med..

[25] Lee K. J., Choi J. H., Jeong H. G. (2007). Hepatoprotective and antioxidant
effects of the coffee diterpenes kahweol and cafestol on carbon tetrachloride-induced
liver damage in mice. Food Chem. Toxicol..

[26] de
Oliveira N. A., Sandini T. M., Cornelio-Santiago H. P., Martinelli E. C. L., Raspantini L. E. R., Raspantini P. C., Momo C., de Oliveira A. L., Fukumasu H. (2020). Acute and subacute
(28 days) toxicity of green coffee oil enriched with diterpenes cafestol
and kahweol in rats. Regul. Toxicol. Pharmacol..

[27] Van
Cruchten S., de Waart D. R., Kunne C., Hooiveld G. J. E. J., Boekschoten M. V., Katan M. B., Elferink R. P. J. O., Witkamp R. F. (2010). Absorption, Distribution, and Biliary Excretion of
Cafestol, a Potent Cholesterol-Elevating Compound in Unfiltered Coffees,
in Mice. Drug Metab. Dispos..

[28] van
Cruchten S. T. J., de Haan L. H. J., Mulder P. P. J., Kunne C., Boekschoten M. V., Katan M. B., Aarts J. M. M. J. G., Witkamp R. F. (2010). The role of epoxidation and electrophile-responsive
element-regulated gene transcription in the potentially beneficial
and harmful effects of the coffee components cafestol and kahweol. J. Nutr. Biochem..

[29] Andriolo C. V., Novaes F. J. M., Pereira H. M. G., Sardela V. F., Rezende C. M. (2021). Metabolic
study of cafestol using in silico approach, zebrafish water tank experiments
and liquid chromatography high-resolution mass spectrometry analyses. J. Chromatogr B Analyt Technol. Biomed Life Sci..

[30] Basile D. P., Anderson M. D., Sutton T. A. (2012). Pathophysiology
of Acute Kidney Injury. Compr. Physiol..

[31] Rangel L. B. A., Lopes A. G., Lara L. S. M., Carvalho T. L. G., Silva I. V., Oliveira M. M., Einicker-Lamas M., Vieyra A., Nogaroli L., Caruso-Neves C. (2005). PI-PLCβ is involved in the modulation of the
proximal tubule Na+-ATPase by angiotensin II. Regul. Pept..

[32] Lara L. S., Correa J. S., Lavelle A. B., Lopes A. G., Caruso-Neves C. (2008). The angiotensin
receptor type 1-Gq protein-phosphatidyl inositol phospholipase Cbeta-protein
kinase C pathway is involved in activation of proximal tubule Na+-ATPase
activity by angiotensin(1–7) in pig kidneys. Experimental physiology.

[33] Nieuwenhuijs-Moeke G.
J., Pischke S. E., Berger S. P., Sanders J. S. F., Pol R. A., Struys M. M. R. F., Ploeg R. J., Leuvenink H. G. D. (2020). Ischemia
and Reperfusion Injury in Kidney Transplantation: Relevant Mechanisms
in Injury and Repair. Journal of Clinical Medicine.

[34] Aragno M., Cutrin J. C., Mastrocola R., Perrelli M.-G., Restivo F., Poli G., Danni O., Boccuzzi G. (2003). Oxidative stress and
kidney dysfunction due to ischemia/reperfusion in rat: Attenuation
by dehydroepiandrosterone. Kidney International.

[35] Fekete A., Vannay Á., Vér Á., Vásárhelyi B., Müller V., Ouyang N., Reusz G., Tulassay T., Szabó A. J. (2004). Sex differences
in the alterations of Na^+^,K^+^-ATPase following
ischaemia-reperfusion injury in the
rat kidney. J. Physiol..

[36] Queiroz-Madeira E. P., Lara L. S., Wengert M., Landgraf S. S., Líbano-Soares J. D., Zapata-Sudo G., Sudo R. T., Takiya C. M., Gomes-Quintana E., Lopes A. G., Caruso-Neves C. (2010). Na­(+)-ATPase in spontaneous hypertensive
rats: possible AT(1) receptor target in the development of hypertension. Biochimica Et Biophysica Acta.

[37] Wang Z., Zhang C. (2022). From AKI to CKD: Maladaptive
Repair and the Underlying Mechanisms. Int. J.
Mol. Sci..

[38] Caron A., Desrosiers R. R., Langlois S., Béliveau R. (2005). Ischemia-reperfusion
injury stimulates gelatinase expression and activity in kidney glomeruli. Can. J. Physiol. Pharmacol..

[39] Sheetal G. S., Peter V. S., Peter (2020). In Vitro Action of Matrix Metalloproteinases
2 and
9 Inhibitors on Na + /K + -ATPase, H + /K + -ATPase and PMCA Activities
in the Osmoregulatory Epithelia of Climbing Perch (Anabas testudineus
Bloch). J. Endocrinol. Reprod..

[40] Basile D. P., Fredrich K., Weihrauch D., Hattan N., Chilian W. M. (2004). Angiostatin
and matrix metalloprotease expression following ischemic acute renal
failure. Am. J. Physiol Renal Physiol.

[41] Lee S. Y., Hörbelt M., Mang H. E., Knipe N. L., Bacallao R. L., Sado Y., Sutton T. A. (2011). MMP-9 gene deletion mitigates microvascular
loss in a model of ischemic acute kidney injury. Am. J. Physiol Renal Physiol.

[42] Chen B., Brem A. S., Gong R. (2022). The Janus view: dual
roles for hypoxia-inducible
factor in renal repair after acute kidney injury. American Journal of Physiology-renal Physiology.

[43] Fang Y., Yu X., Liu Y., Kriegel A. J., Heng Y., Xu X., Liang M., Ding X. (2013). miR-29c is downregulated in renal
interstitial fibrosis in humans and rats and restored by HIF-α
activation. Am. J. Physiol Renal Physiol.

[44] Kobayashi H., Gilbert V., Liu Q., Kapitsinou P. P., Unger T. L., Rha J., Rivella S., Schlöndorff D., Haase V. H. (2012). Myeloid cell-derived hypoxia-inducible
factor attenuates
inflammation in unilateral ureteral obstruction-induced kidney injury. J. Immunology.

[45] Shu S., Wang Y., Zheng M., Liu Z., Cai J., Tang C., Dong Z. (2019). Hypoxia and Hypoxia-Inducible Factors
in Kidney Injury and Repair. Cells.

[46] Jamadarkhana P., Chaudhary A., Chhipa L., Dubey A., Mohanan A., Gupta R., Deshpande S. (2012). Treatment with a novel hypoxia-inducible
factor hydroxylase inhibitor (TRC160334) ameliorates ischemic acute
kidney injury. Am. J. Nephrol.

[47] Novaes F. J. M., Lima F. A., Calado V., Marriott P. J., de Aquino
Neto F. R., Rezende C. M. (2020). Isolating valuable coffee diterpenes
by using an inexpensive procedure. Industrial
Crops and Products.

[48] Lowry O. H., Rosebrough N. J., Farr A. L., Randall R. J. (1951). Protein
measurement
with the Folin phenol reagent. J. Biol. Chem..

[49] Pereira-Acácio A., Veloso-Santos J. P. M., Nossar L. F., Costa-Sarmento G., Muzi-Filho H., Vieyra A., Cheng X. (2022). Angiotensin-(3–4)
normalizes the elevated arterial blood pressure and abnormal Na+/energy
handling associated with chronic undernutrition by counteracting the
effects mediated by type 1 angiotensin II receptors. PLoS One.

[50] Beiral H. J. V., Rodrigues-Ferreira C., Fernandes A. M., Gonsalez S. R., Mortari N. C., Takiya C. M., Sorenson M. M., Figueiredo-Freitas C., Galina A., Vieyra A. (2014). The Impact of Stem
Cells on Electron Fluxes, Proton Translocation, and ATP Synthesis
in Kidney Mitochondria after Ischemia/Reperfusion. Cell Transplant..

[51] Radulović N. S., Randjelović P. J., Stojanović N. M., Ilić I. R., Miltojević A.
B., Stojković M. B., Ilić M. (2015). Effect of two esters of N-methylanthranilic acid from
Rutaceae species on impaired kidney morphology and function in rats
caused by CCl4. Life Sciences.

[52] Bedoya S. A. O., Souza M. V., Conceição L. G., Viloria M. I. V., Valente F. L., Loures F. H., Moreira J. C. L., Coelho P. G. B. (2019). Quantificação
do colágeno dérmico equino por duas técnicas
morfométricas: contagem de pontos e segmentação
de cor. Arq. Bras. Med. Vet. Zootec..

[53] Heussen C., Dowdle E. B. (1980). Electrophoretic analysis of plasminogen
activators
in polyacrylamide gels containing sodium dodecyl sulfate and copolymerized
substrates. Anal. Biochem..

