# Supplementary material for: Pharmacological Potential of Cafestol, a Bioactive Substance in Coffee, in Preventing Ischemia-Reperfusion-Induced Acute Kidney Injury
Source: ACS Omega. 2025 May 27;10(22):22825–36. doi: 10.1021/acsomega.4c11728 (PMC12163779; doi:10.1021/acsomega.4c11728)
Supplement: Supplementary file 1 [file ao4c11728_si_001.pdf]

Pharmacological potential of cafestol, a bioactive substance in coffee, in preventing ischemia-reperfusion-induced acute kidney injury

Dayene S. Gomes[a,#] Mayara A. Romanelli[a,#], Stela P.S. Gomes[a], Ana Laura M. Brand[b], Rodrigo M.V. da Silva[b], Simone S.C. Oliveira[c], André Luis S. Santos[c], Claudia M. Rezende[b], Lucienne S. Lara[a,*]

[a]Instituto de Ciências Biomédicas and Centro de Pesquisa em Medicina de Precisão, Universidade Federal do Rio de Janeiro, Rio de Janeiro, 21941-902, Brazil

[b]Instituto de Química, Centro de Ciências Matemáticas e da Natureza, Universidade Federal do Rio de Janeiro, Rio de Janeiro, 21941-909, Brazil

[c] Instituto de Microbiologia Paulo de Góes, Universidade Federal do Rio de Janeiro, Rio de Janeiro, 21941-902, Brazil

[#]These authors contributed equally to this work

[*]Corresponding author

Lucienne S. Lara, PhD
Associate Professor
Universidade Federal do Rio de Janeiro
Centro de Ciências da Saúde
Instituto de Ciências Biomédicas
Avenida Carlos Chagas Filho 373, bloco J, sala 26
Rio de Janeiro, RJ, Brazil 21941-902
Phone: +55 21 39386733
Email: lara@icb.ufrj.br or lucienne.morcillo@gmail.com

Supplementary information

Purity of cafestol extraction

Cafestol ($C_{20}H_{28}O_3$): white solid; m.p. 153.5–154.0 °C; 1H NMR (500 MHz, CDCl3, δ ppm): 7.25 (1H, d, J = 1.8 Hz, H19), 6.22 (1H, d, J = 1.8 Hz, H18), 5.31 (1H, s, C16–OH), 3.82 (1H, d, J = 11.1 Hz, H17a), 3.70 (1H, d, J = 11.1 Hz, H17b), 2.62 (2H, dd, J = 5.8, 2.7 Hz, H2), 2.27 (1H, dq, J = 12.6, 2.7 Hz, H5), 2.07–2.03 (3H, m, H13, H1, H14), 1.82 (1H, ddd, J = 12.9, 6.2, 3.1 Hz, 455 H6), 1.74–1.51 (10H, m, H6, H7, H11, H12, H14, H15), 1.27–1.23 (1H, m, H1), 1.19 (1H, d, J = 7.7 Hz, H9), 0.84 (3H, s, H20). 13C NMR (500 MHz, CDCl3, δ ppm): 148.8 (C, C3), 457140.7 (CH, C19), 120.2 (C, C4), 108.4 (CH, C18), 82.1 (C, C16), 66.4 (CH2, C17), 53.4458 (CH2, C15), 52.2 (CH, C9), 45.5 (CH, C13), 44.8 (C, C8), 44.3 (CH, C5), 41.0 (CH2, C7), 38.7 (C, C10), 38.3 (CH2, C14), 35.8 (CH2, C1), 26.2 (CH2, C12), 23.2 (CH2, C6), 20.8 (CH2, C2), 19.1 (CH2, C11), 13.5 (CH3, C20). HRMS (ESI+) for protonated cafestol ($C_{20}H_{29}O_3$). Exact Mass: $[M+H]^+$ 17.2111, found 317.21057 (error 1.67 ppm). All the results are in accordance with the literature [1, 2].

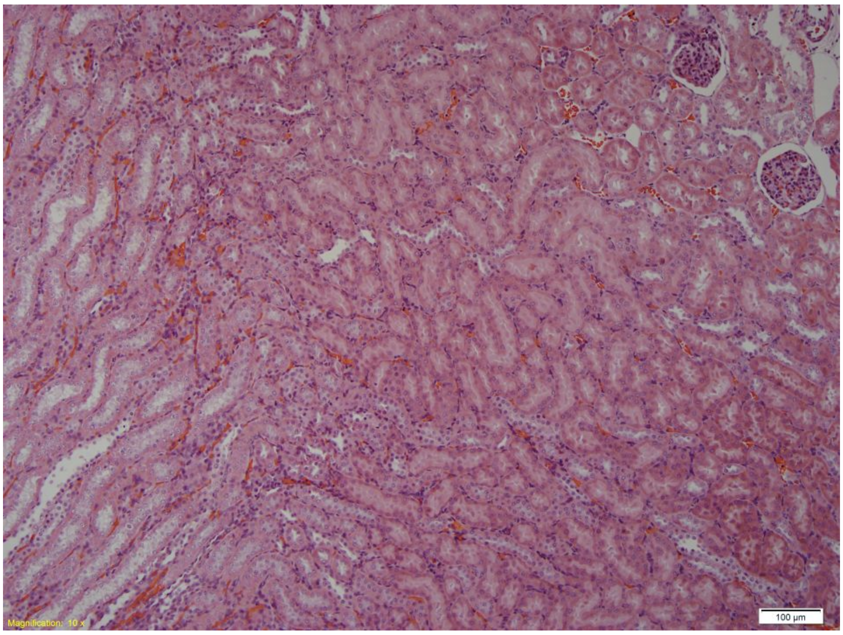

Figure S1: Histological image of a kidney from a typical rat (with no vehicle administration nor submitted to a surgery process). The kidneys presented normal morphology, with distinguishable characteristics of proximal and distal tubule. The glomerulus presented a typical appearance

References

[1] F.J.M. Novaes, F.A. Lima, V. Calado, P.J. Marriott, F.R. de Aquino Neto, C.M. Rezende, Isolating valuable coffee diterpenes by using an inexpensive procedure, Ind Crops Prod. 152 (2020) 112494. https://doi.org/10.1016/j.indcrop.2020.112494.

[2] F.A. Lima, M.A.M. Bezerra, R. Souza, I. Itabaiana, T. Haynes, S. Hermans, R. Wojcieszak, F.Junior M. Novaes, C.M. Rezende, Fast and Highly Selective Continuous-Flow Catalytic Hydrogenation of a Cafestol–Kahweol Mixture Obtained from Green Coffee Beans, ACS Omega. 5 (2020) 25712–25722. https://doi.org/10.1021/acsomega.0c01835.
